# Supplementary figures and images for: Seed size and its rate of evolution correlate with species diversification across angiosperms
Source: PLoS Biol. 2017 Jul 19;15(7):e2002792. doi: 10.1371/journal.pbio.2002792 (PMC5536390; doi:10.1371/journal.pbio.2002792)

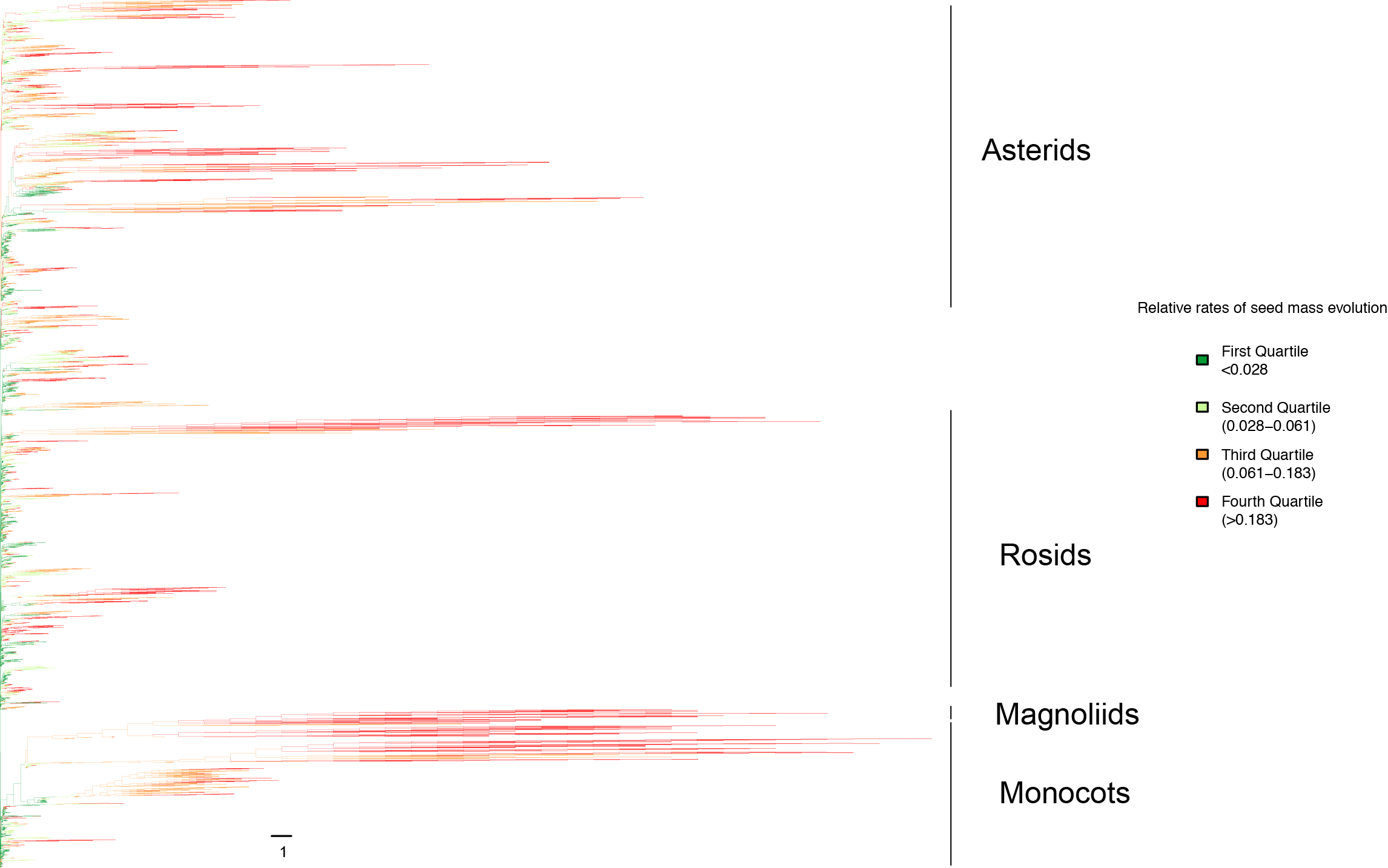

Supplement: S1 Fig — Branches were scaled by speciation rate as determined by a BAMM analysis on a larger 19,703 tree. (TIF) [file pbio.2002792.s001.tif]

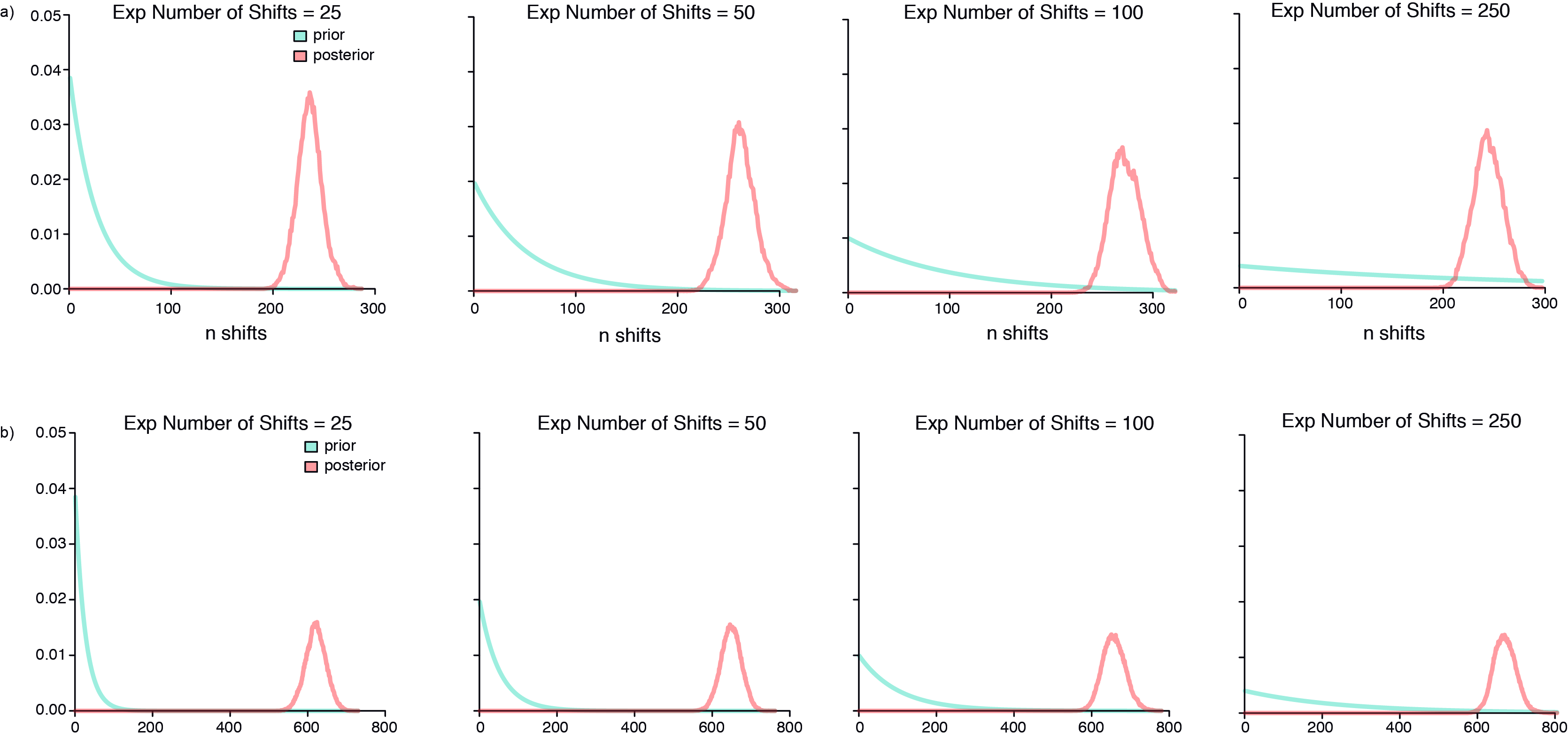

Supplement: S2 Fig — a) the speciation/extinction and b) phenotypic evolution analyses for expectedNumberOfShifts = 25, 50 and 100 and 250. The analyses in the main text were carried out with expectedNumberOfShifts = 50 for both speciation/extinction and phenotypic evolution analyses. (TIF) [file pbio.2002792.s002.tif]

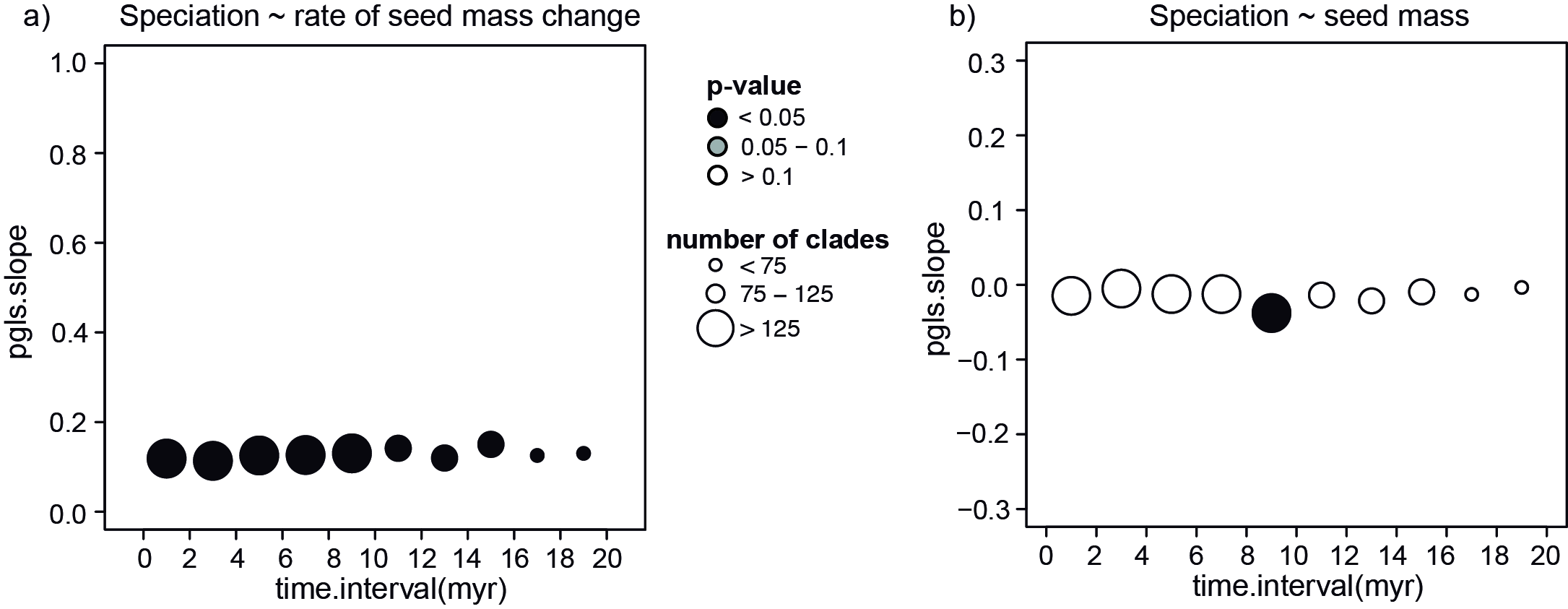

Supplement: S3 Fig — (a) PGLS slope of the relationship between speciation rate (λ) from the method-of-moments estimator and the rate of seed mass evolution across 10 time slices. Circles are scaled to the number of clades in each time slice while colour indicates the significance of the slope. (b) PGLS slope of the relationship between speciation rate and mean clade seed mass. For a detailed representation of the results in each time slice, see S14 and S15 Figs. (TIF) [file pbio.2002792.s003.tif]

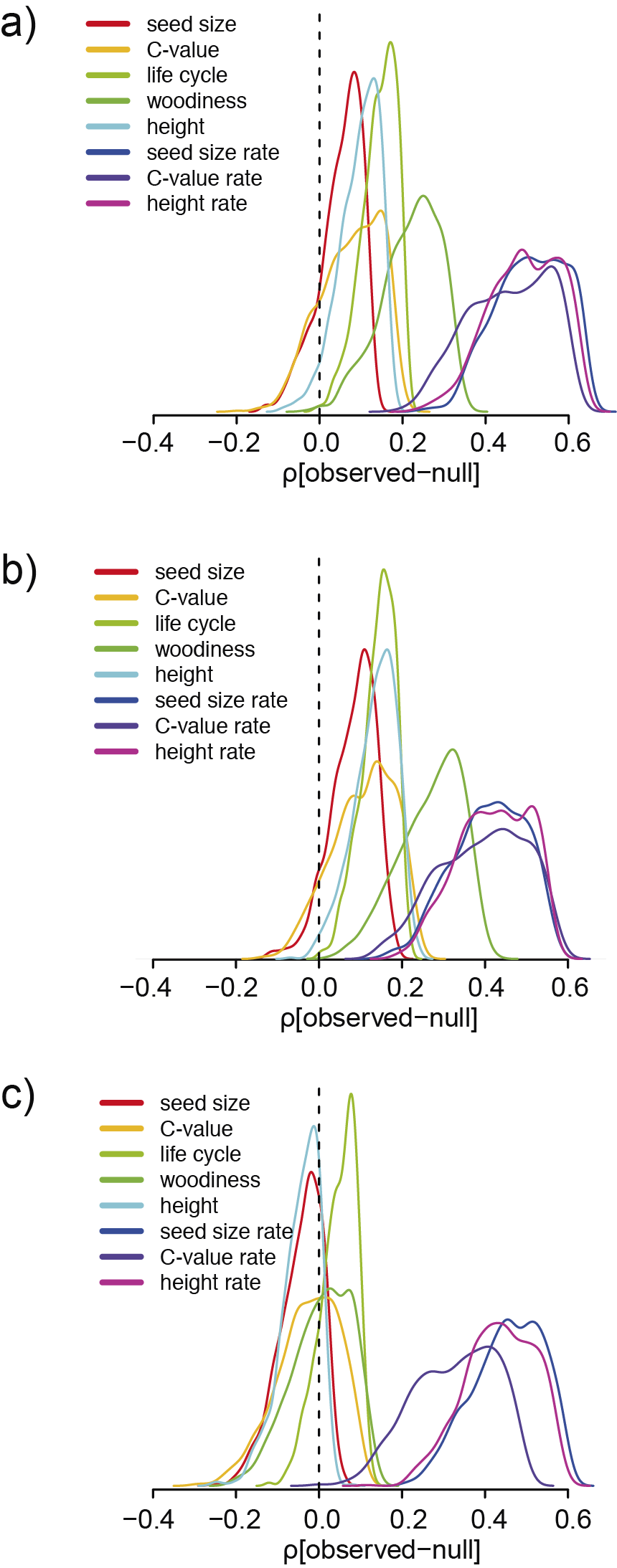

Supplement: S4 Fig — The distribution of the absolute difference in the observed correlation minus the null correlation is plotted for each trait. The coloured dotted lines indicate the mean of that distribution, and the black dotted line indicates 0; a distribution with mean = 0 would show no association between a focal trait and macroevolutionary dynamics. STRAPP correlation of seed size (shown in red), C-value (shown in yellow), life cycle (shown in light green), woodiness (shown in dark green), height (shown in blue), seed size rate (shown in dark blue), C-value rate (shown in purple); and height rate (shown in pink) with a) speciation rate (λ), b) extinction rate (μ), and c) net diversification rate (r). (TIF) [file pbio.2002792.s004.tif]

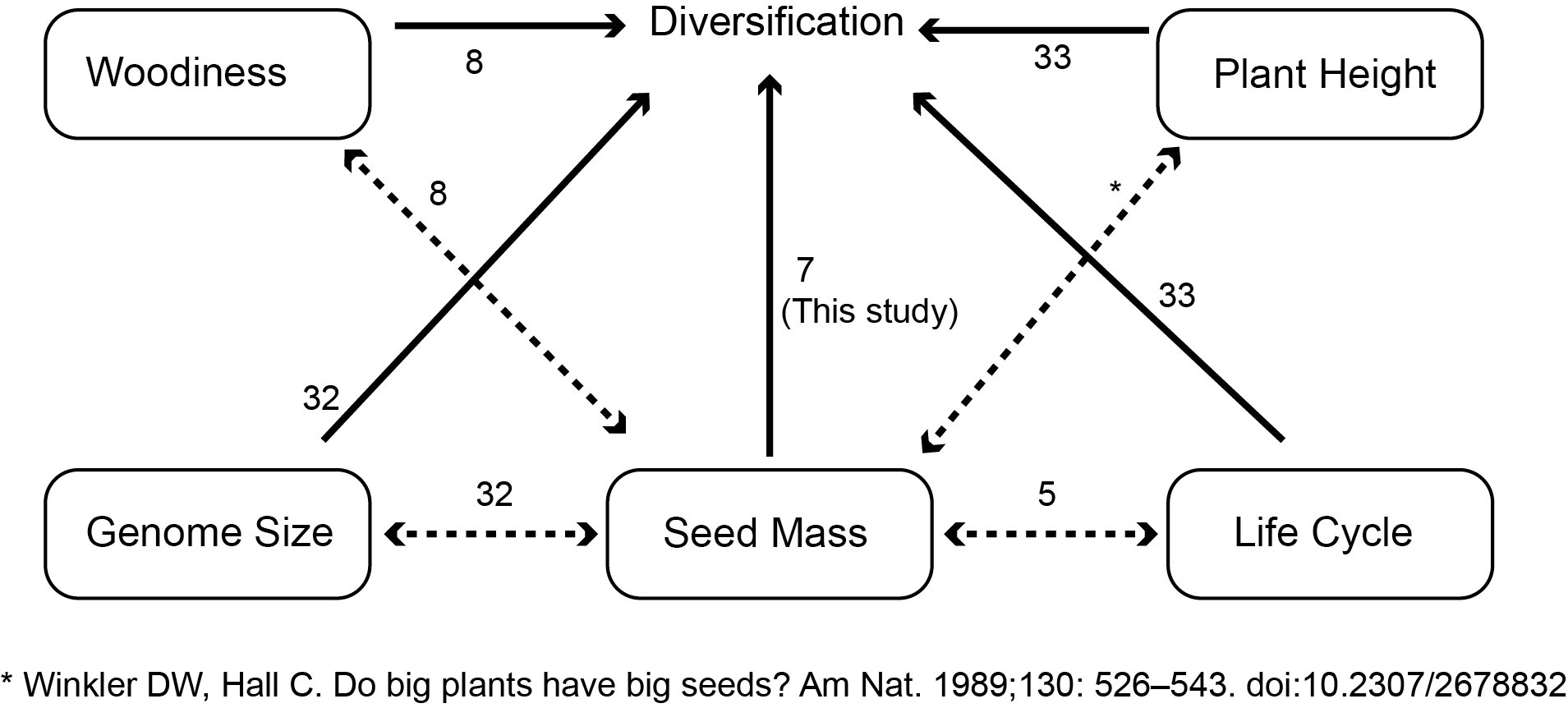

Supplement: S5 Fig — (Solid lines). Dashed lines indicate correlations between life history traits. Numbers indicate reference where the link is proposed. (TIF) [file pbio.2002792.s005.tif]

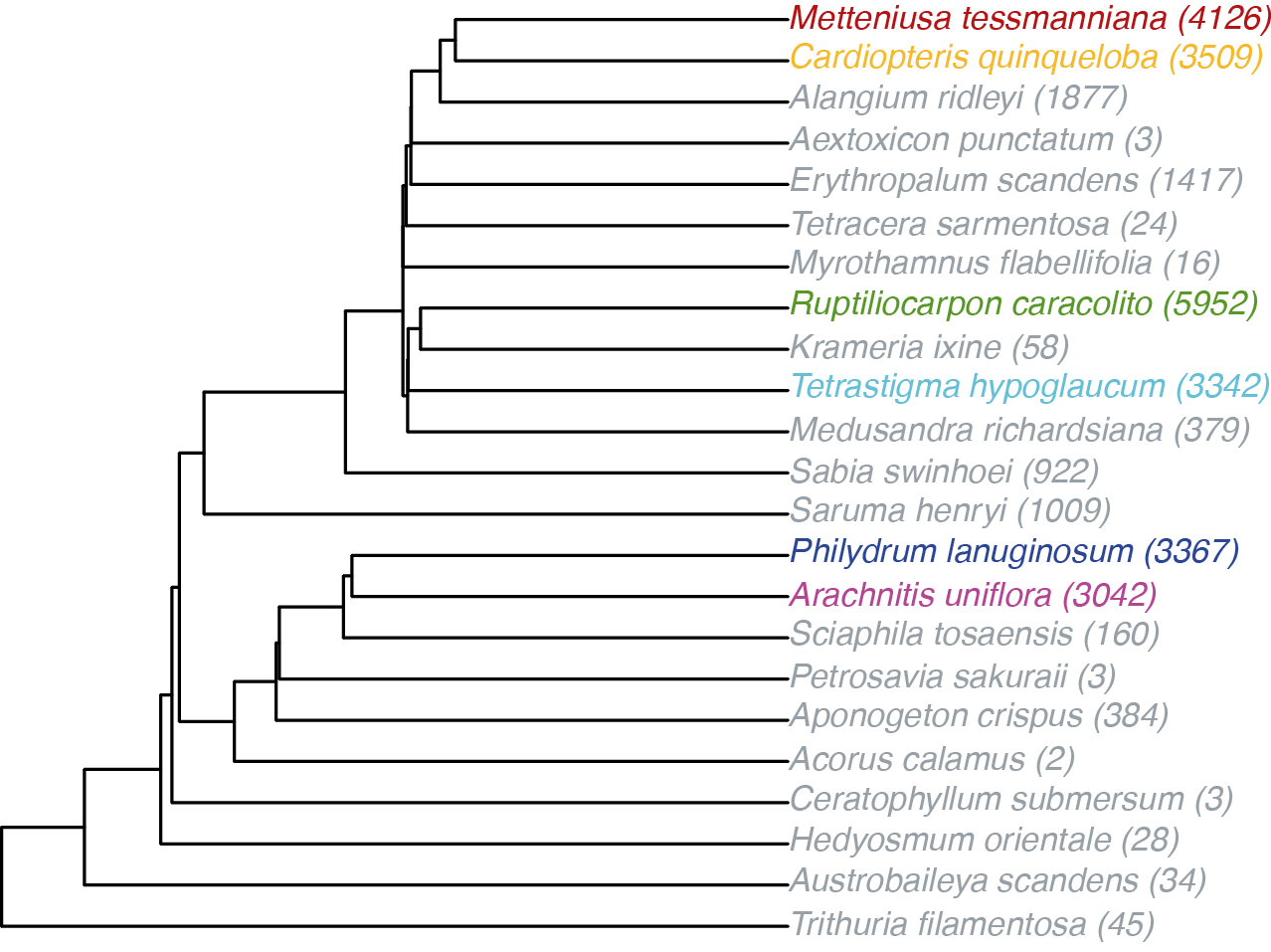

Supplement: S6 Fig — The red bars indicate the levels of sampling for each family. (TIF) [file pbio.2002792.s006.tif]

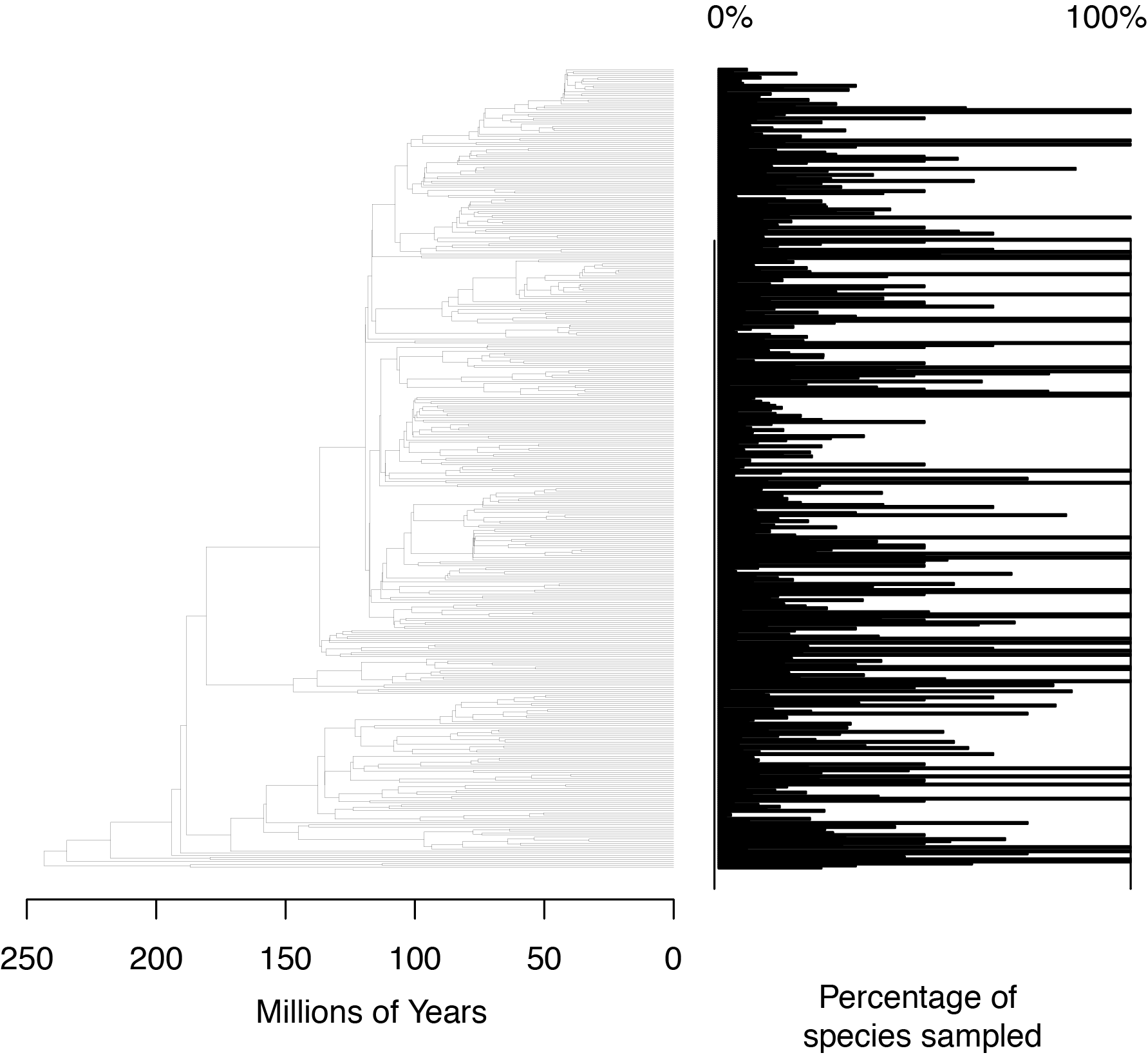

Supplement: S7 Fig — The name of one representative species per clade is shown, and the numbers in parentheses indicate the number of species included in each clade. The BAMM analyses were carried out for six monophyletic clades (shown in red, yellow, green, blue, dark blue and pink) and one “backbone” analysis with the remaining clades (shown in grey) and one representative of each of the six monophyletic clades. (TIF) [file pbio.2002792.s007.tif]

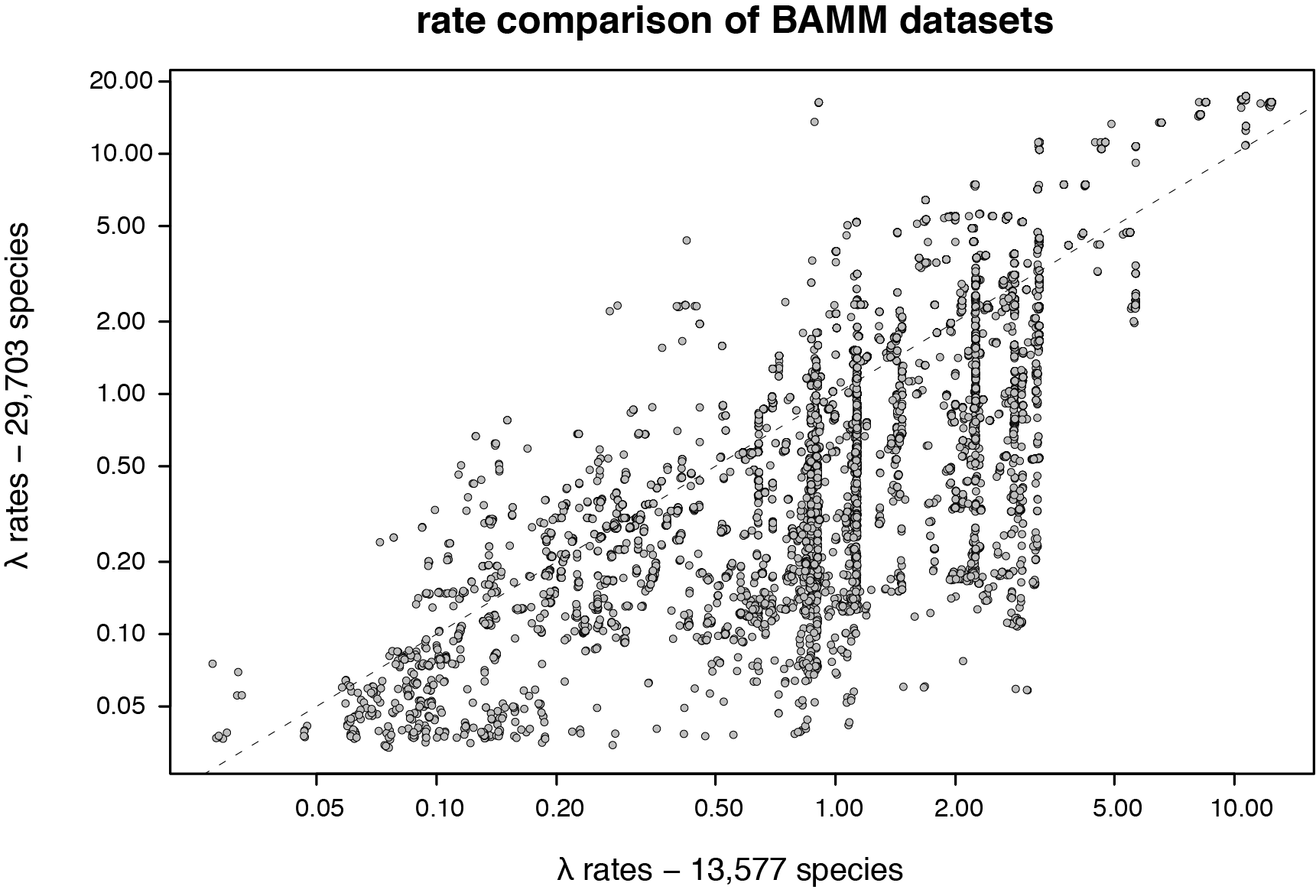

Supplement: S8 Fig — The dotted line represents the 1:1 reference line. (TIF) [file pbio.2002792.s008.tif]

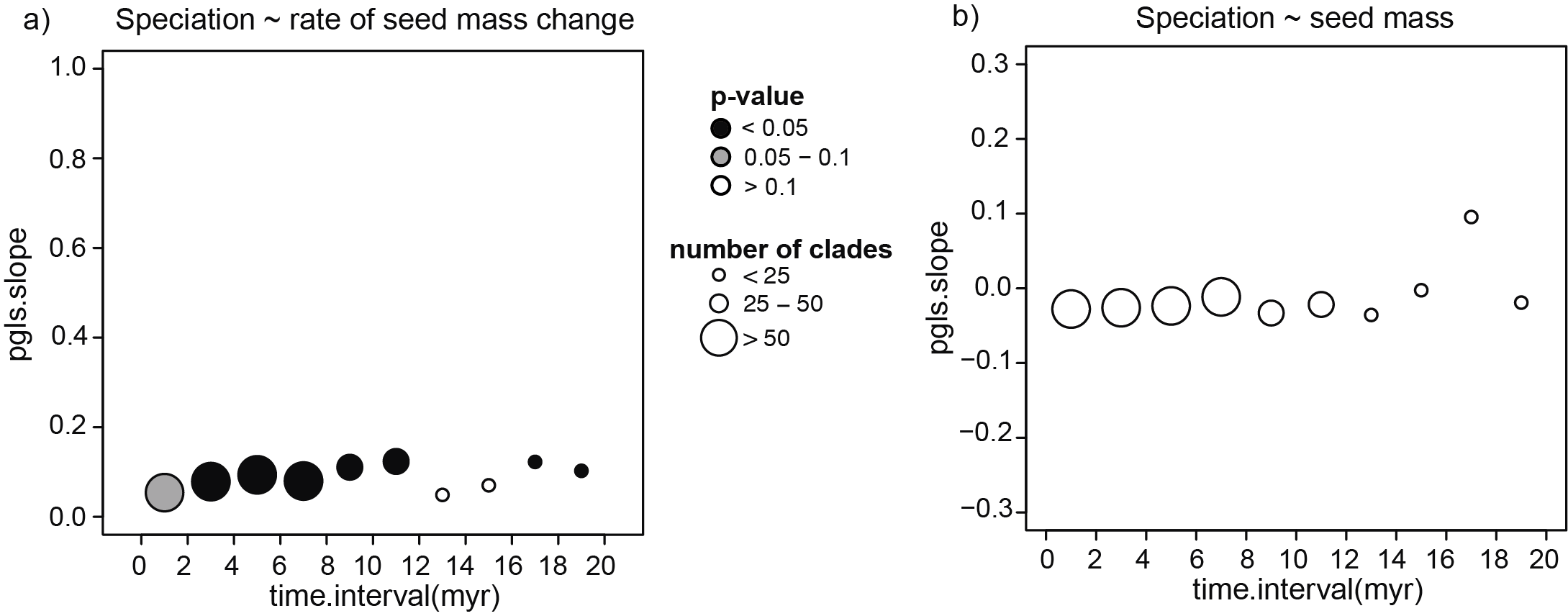

Supplement: S9 Fig — (a) PGLS slope of the relationship of speciation rate—estimated with the method-of-moments estimator—with mean clade seed mass across 10 time slices. The size of the circles represents the number of clades in each time slice while the colour indicates the significance of the slope. (b) PGLS slope of the relationship of speciation rate and the rate of seed mass evolution. (TIF) [file pbio.2002792.s009.tif]

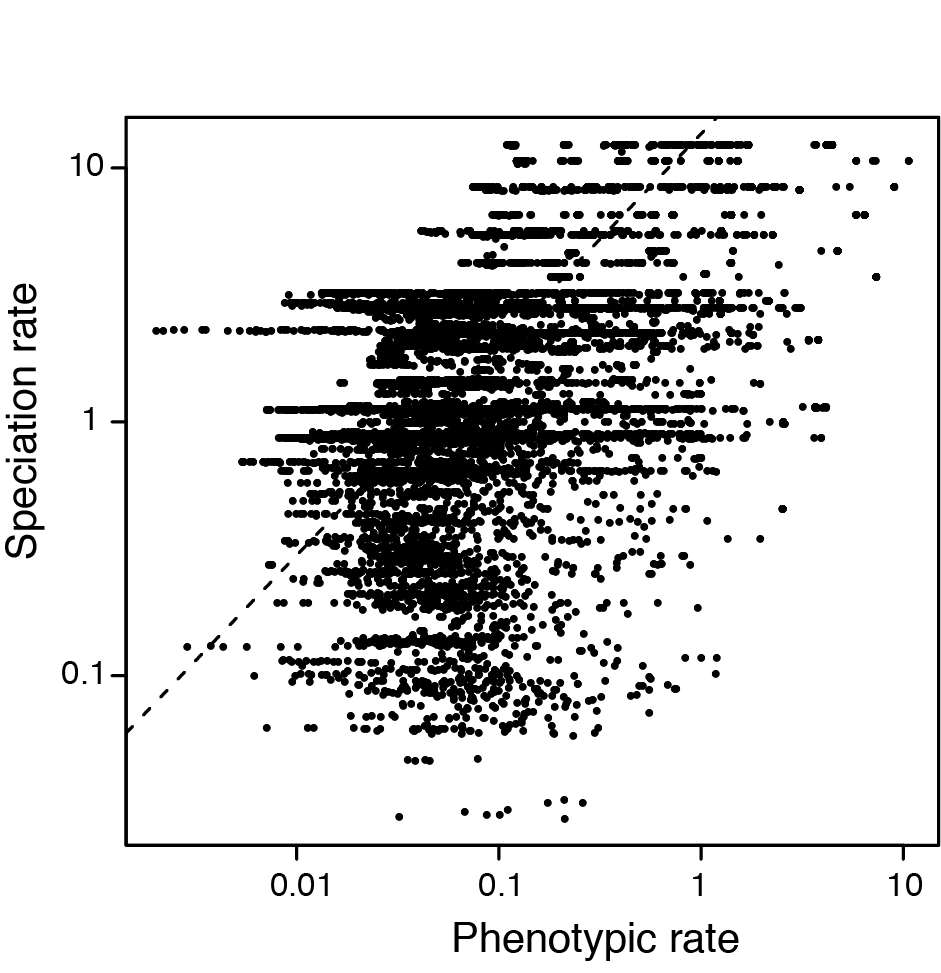

Supplement: S10 Fig — The dotted line represents the Spearman correlation (ρ = 0.47, p-value < 0.001). (TIF) [file pbio.2002792.s010.tif]

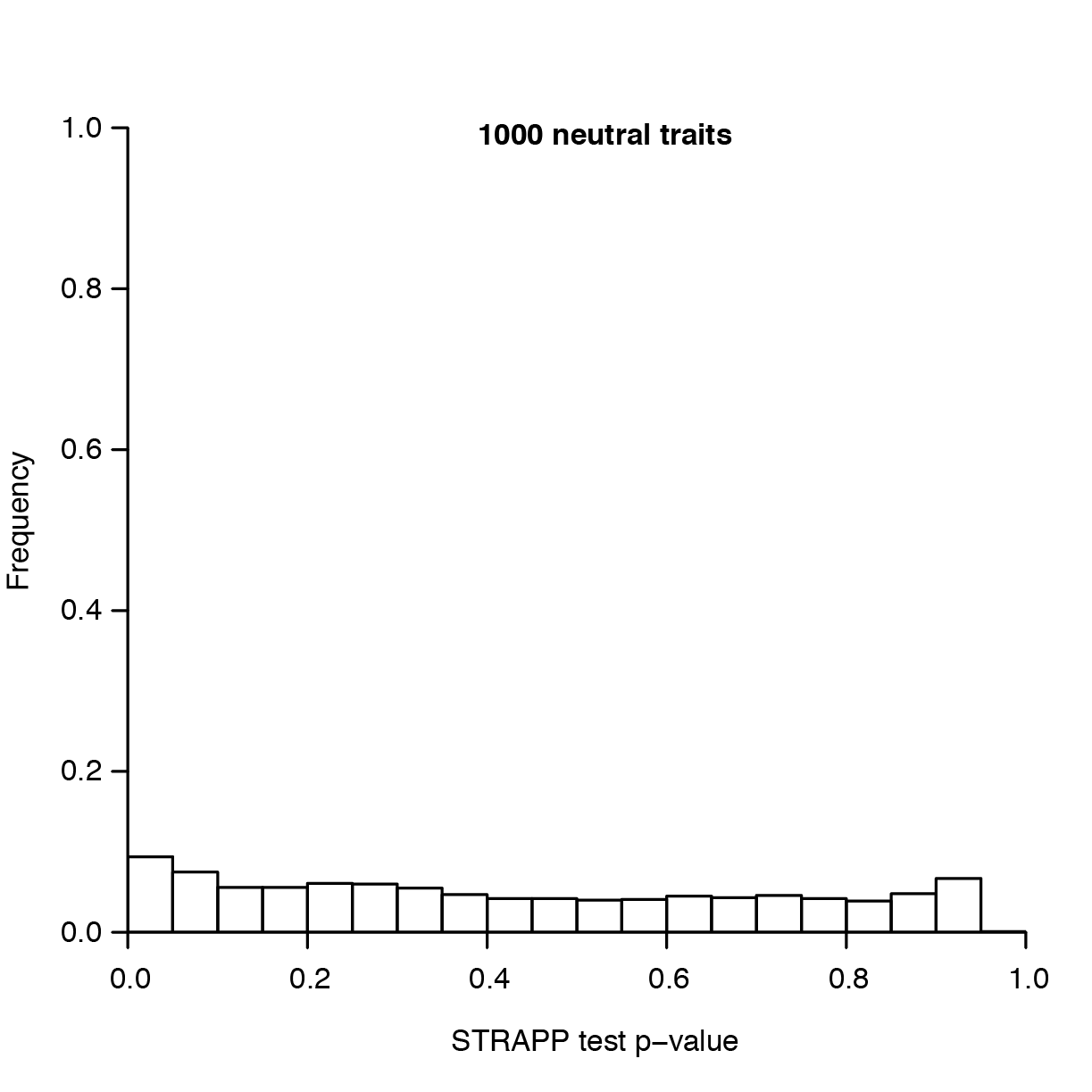

Supplement: S11 Fig — We estimated the type I error rate of our analysis by simulating neutral traits on the angiosperm phylogenetic tree. We performed 1,000 simulations and then ran 1,000 STRAPP tests with each simulated dataset. We estimated the corresponding p-values for the association between traits and diversification and calculated the type I error as the proportion of datasets where a significant association (p-value < 0.05) was detected. (TIF) [file pbio.2002792.s011.tif]

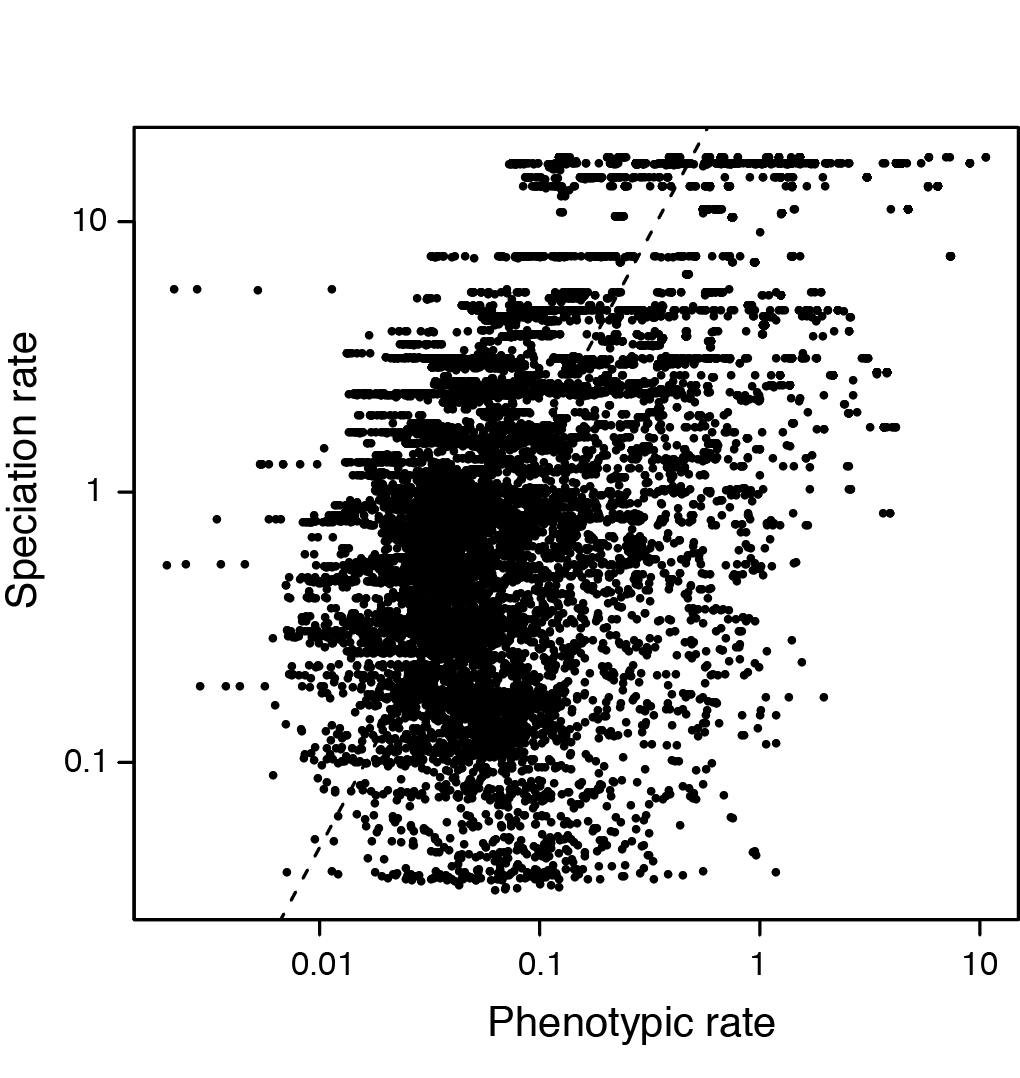

Supplement: S12 Fig — The dotted line is the ordinary least squares regression (R2 = 0.31, p-value < 0.001). (TIF) [file pbio.2002792.s012.tif]

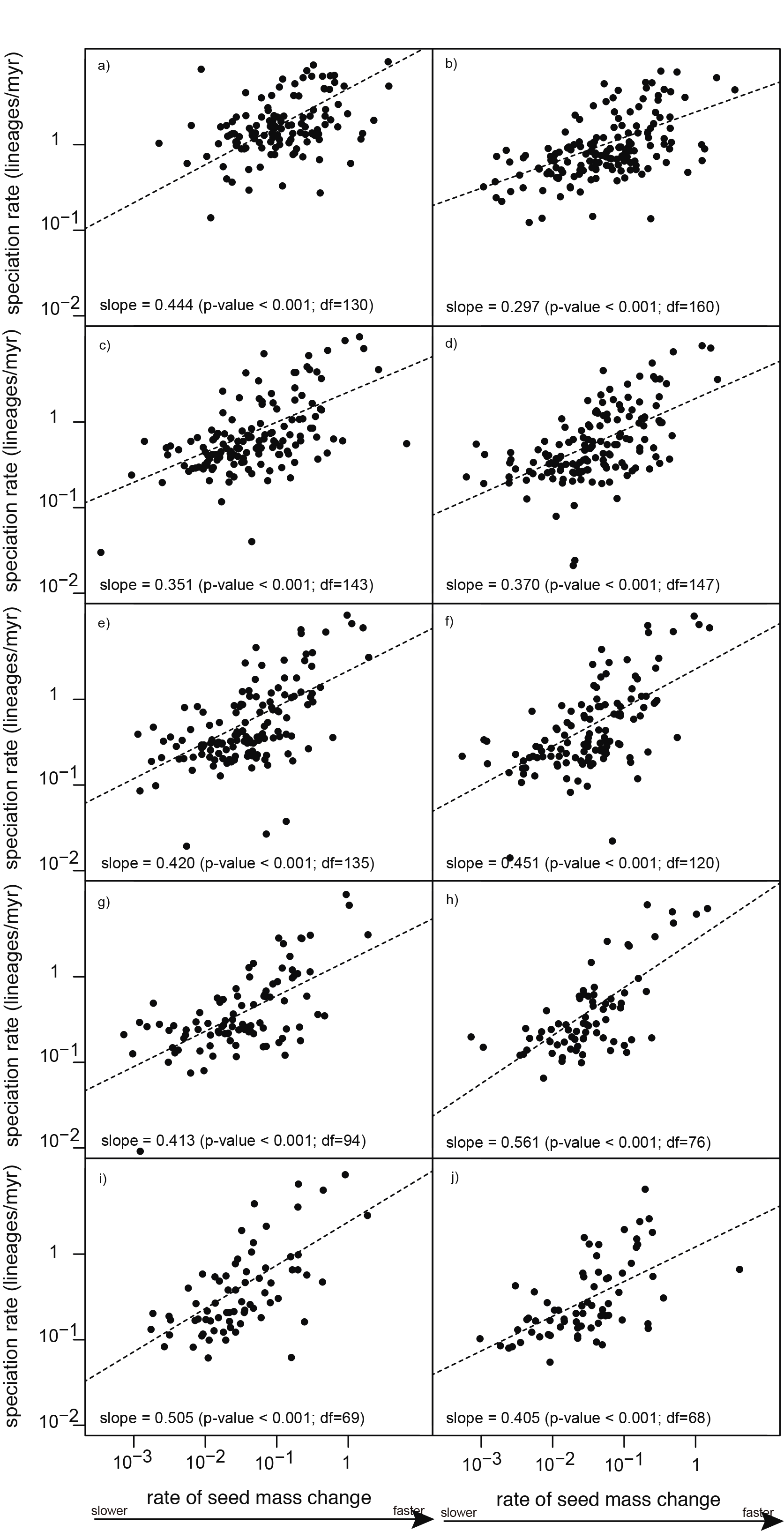

Supplement: S13 Fig — (a) 0 to 2 million years (myr); (b) 2 to 4 myr; (c) 4 to 6 myr; (d) 6 to 8 myr; (e) 8 to 10 myr; (f) 10 to 12 myr; (g) 12 to 14 myr; (h) 14 to 16 myr; (i) 16 to 18 myr; and (j) 18 to 20 myr. The degrees of freedom (df) are equivalent to the number of clades minus one. (TIF) [file pbio.2002792.s013.tif]

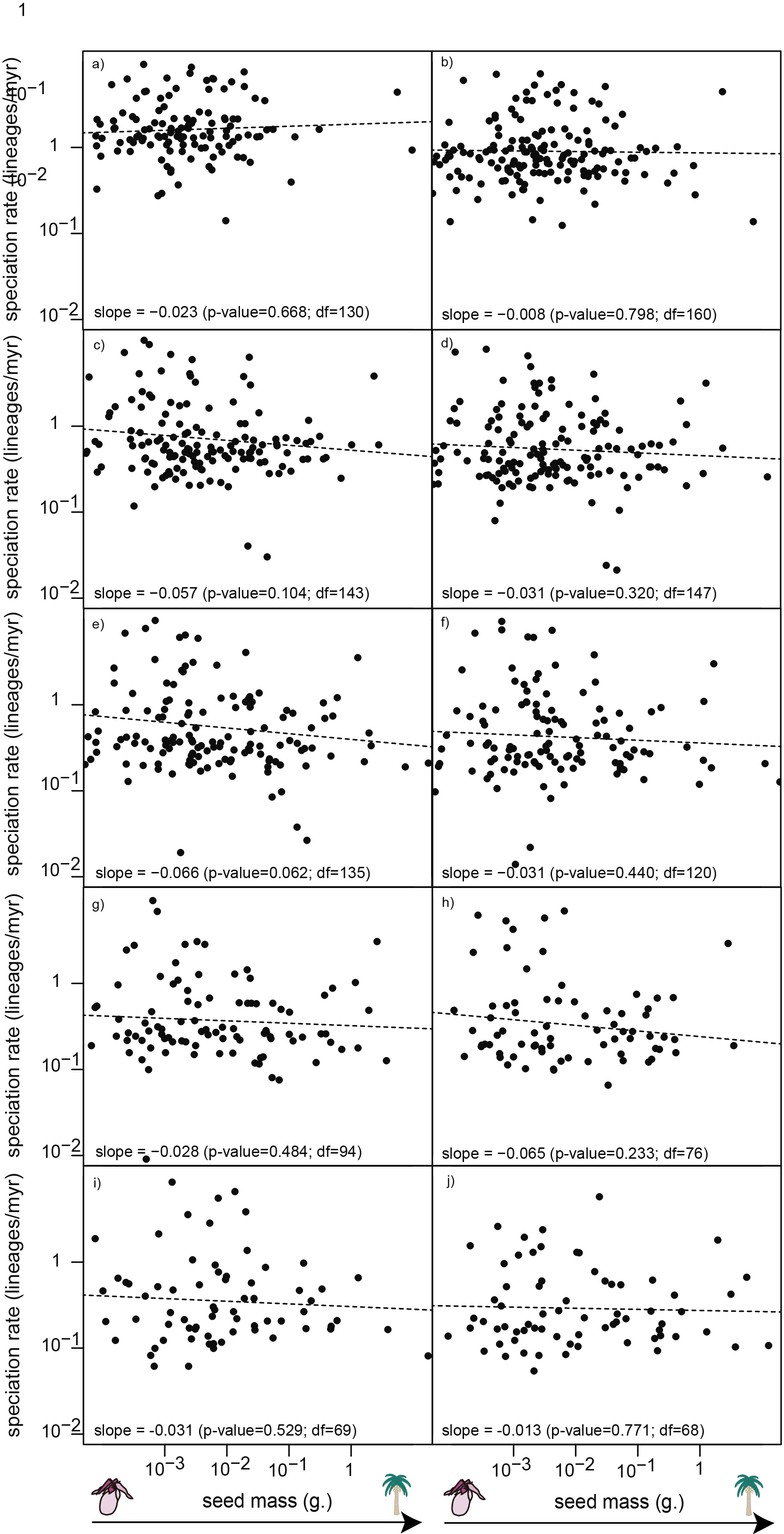

Supplement: S14 Fig — (a) 0 to 2 million years (myr); (b) 2 to 4 myr; (c) 4 to 6 myr; (d) 6 to 8 myr; (e) 8 to 10 myr; (f) 10 to 12 myr; (g) 12 to 14 myr; (h) 14 to 16 myr; (i) 16 to 18 myr; and (j) 18 to 20 myr. The degrees of freedom (df) are equivalent to the number of clades minus one. (TIF) [file pbio.2002792.s014.tif]

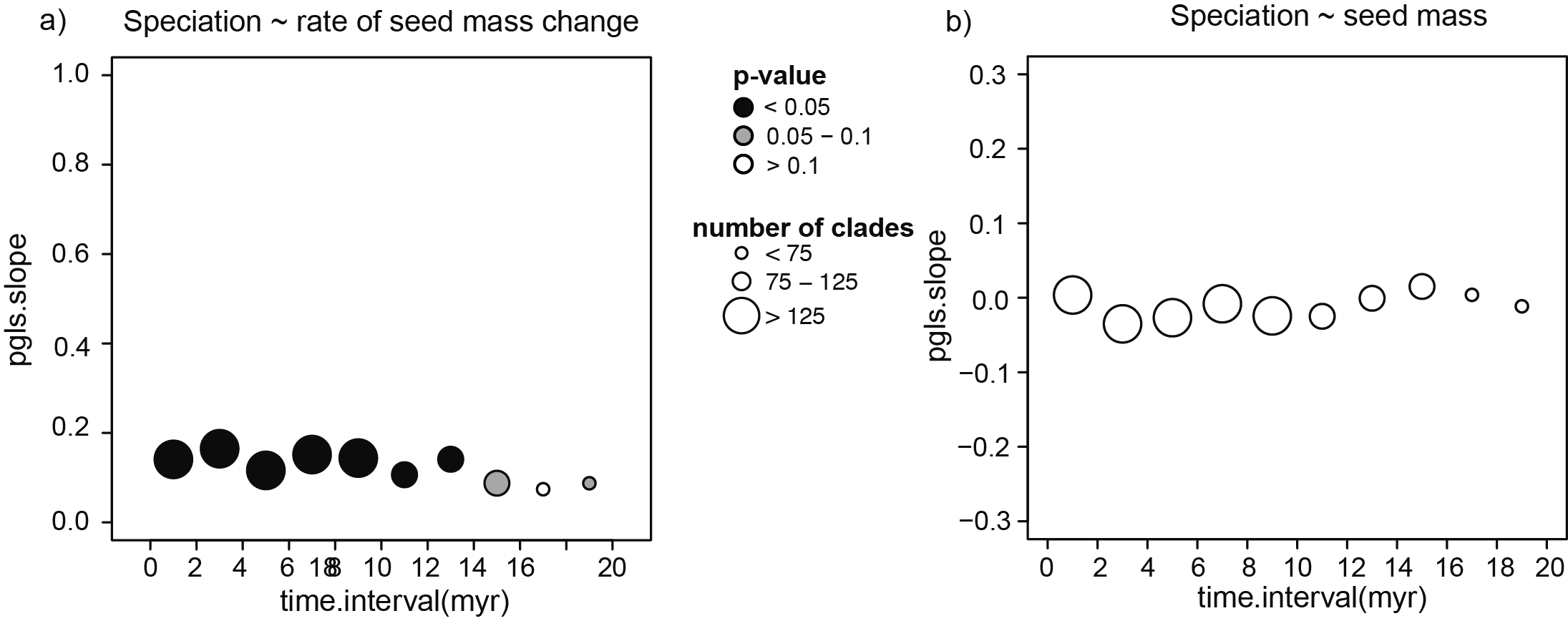

Supplement: S15 Fig — Correlation of (a) rate of seed mass evolution and (b) seed mass with net diversification rate (r) estimated using RPANDA in the clade-based analysis. The strength of correlations is shown as PGLS slopes and was calculated using mean clade-level seed mass across 10 time slices. The size of the circles represents the number of clades in each time slice while the colour indicates the significance of the slope. (TIF) [file pbio.2002792.s015.tif]

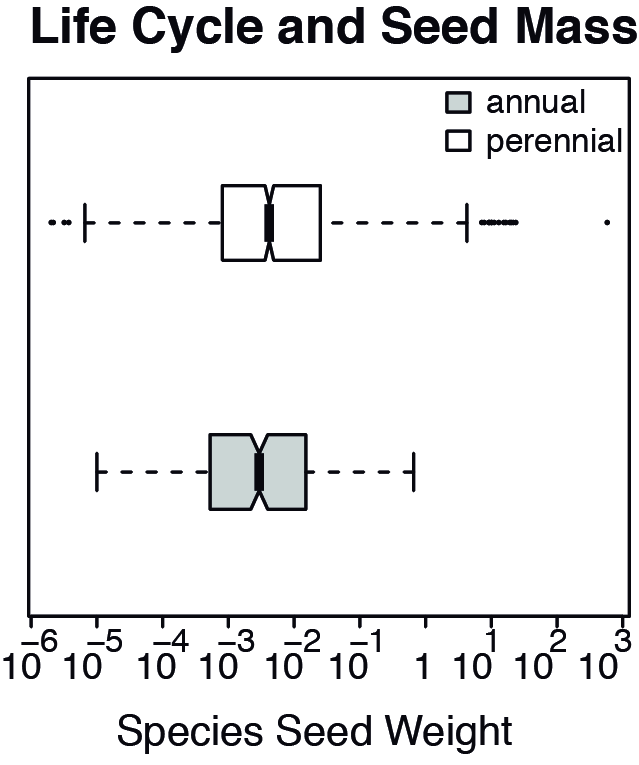

Supplement: S16 Fig — No significant difference between the means of the two groups was found when accounting for phylogeny (phylANOVA: p-value = 0.308, significance assessed with 1,000 random simulations). (TIF) [file pbio.2002792.s016.tif]

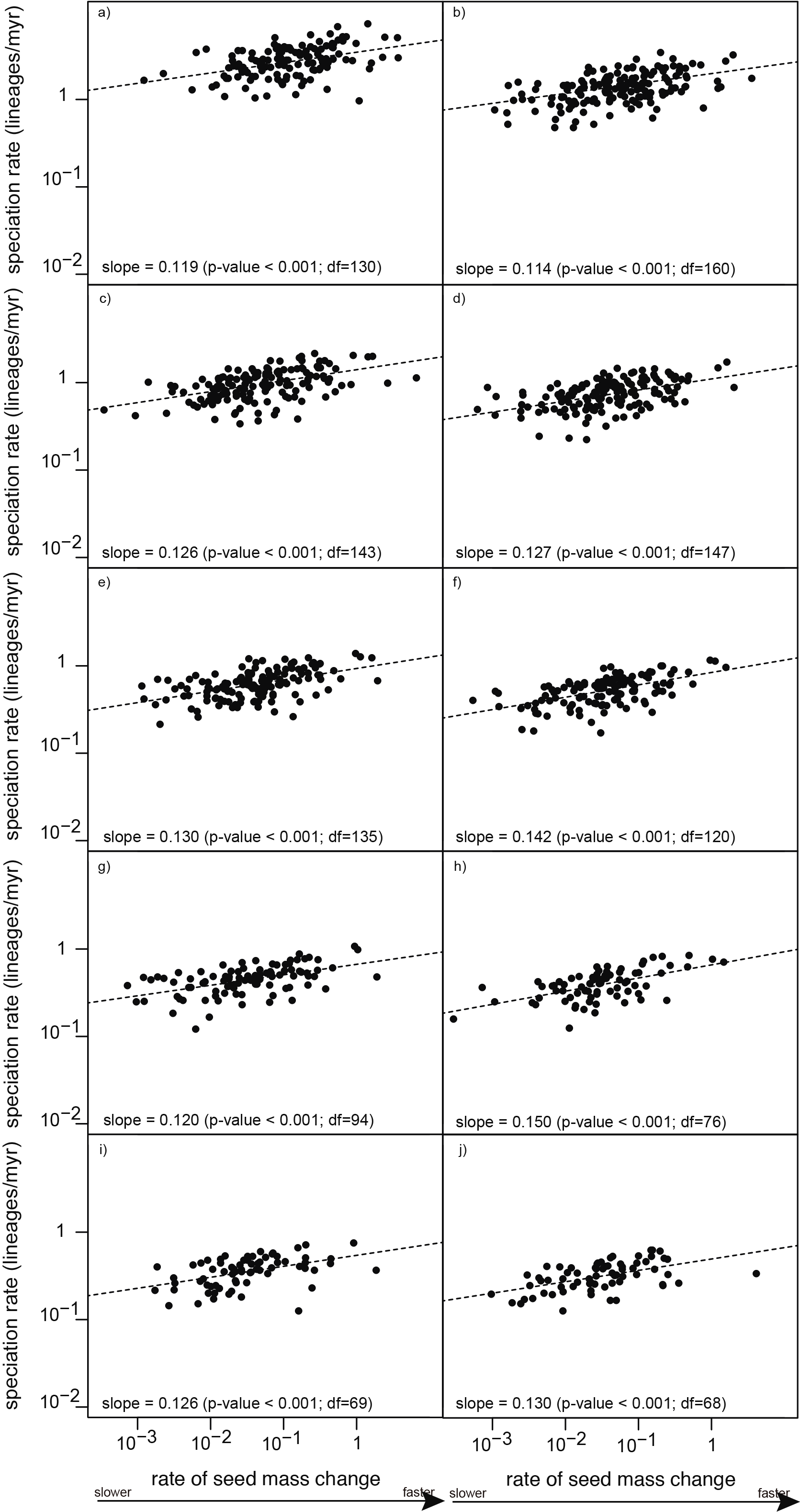

Supplement: S17 Fig — (a) 0 to 2 million years (myr); (b) 2 to 4 myr; (c) 4 to 6 myr; (d) 6 to 8 myr; (e) 8 to 10 myr; (f) 10 to 12 myr; (g) 12 to 14 myr; (h) 14 to 16 myr; (i) 16 to 18 myr; and (j) 18 to 20 myr. (TIF) [file pbio.2002792.s017.tif]

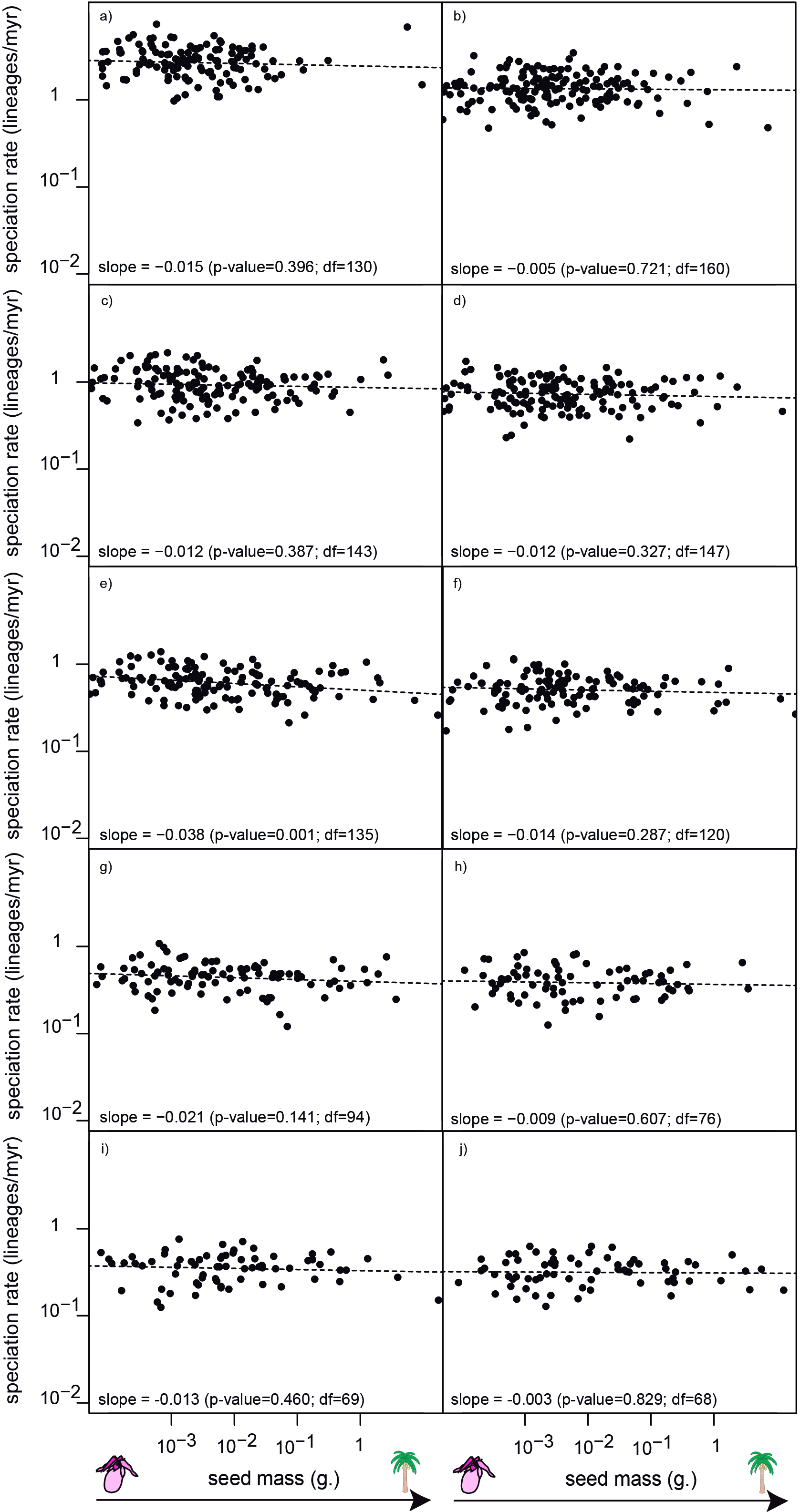

Supplement: S18 Fig — (a) 0 to 2 million years (myr); (b) 2 to 4 myr; (c) 4 to 6 myr; (d) 6 to 8 myr; (e) 8 to 10 myr; (f) 10 to 12 myr; (g) 12 to 14 myr; (h) 14 to 16 myr; (i) 16 to 18 myr; and (j) 18 to 20 myr. (TIF) [file pbio.2002792.s018.tif]
