# Supplementary material for: Seed size and its rate of evolution correlate with species diversification across angiosperms
Source: PLoS Biol. 2017 Jul 19;15(7):e2002792. doi: 10.1371/journal.pbio.2002792 (PMC5536390; doi:10.1371/journal.pbio.2002792)
Supplement: S1 Table — For each 2-million year (myr) time slice, we counted the number of clades where the best-fitting model was either i) birth-death model with constant λ (speciation) and μ (extinction) (lambda.cst.mu.cst); pure birth model with constant λ (lambda.cst.mu0); pure birth model with exponential λ (lambda.exp.mu.0); birth-death model with exponential λ and constant μ (lambda.exp.mu.cst); birth-death model with exponential λ and exponential μ (lambda.exp.mu.exp); or birth-death model with constant λ and exponential μ (lambda.cst.mu.exp). (DOCX) [file pbio.2002792.s019.docx]

| Time slice (myr) | Number of clades | lambda.cst.mu.cst | lambda.cst.mu0 | lambda.exp.mu.0 | lambda.exp.mu.cst | lambda.exp.mu.exp | lambda.cst.mu.exp |
| --- | --- | --- | --- | --- | --- | --- | --- |
| 0-2 | 131 | 7 | 85 | 20 | 2 | 17 | 0 |
| 2-4 | 161 | 14 | 112 | 21 | 0 | 13 | 1 |
| 4-6 | 144 | 20 | 102 | 13 | 0 | 6 | 3 |
| 6-8 | 148 | 21 | 93 | 24 | 0 | 6 | 4 |
| 8-10 | 136 | 20 | 81 | 21 | 2 | 7 | 5 |
| 10-12 | 121 | 21 | 69 | 21 | 1 | 5 | 4 |
| 12-14 | 95 | 12 | 54 | 18 | 1 | 6 | 4 |
| 14-16 | 77 | 10 | 44 | 15 | 0 | 3 | 5 |
| 16-18 | 70 | 15 | 38 | 12 | 0 | 4 | 1 |
| 18-20 | 69 | 10 | 43 | 10 | 1 | 0 | 5 |
| Total | 1152 | 150 (13.02%) | 721(62.59%) | 175(15.19%) | 7(0.61%) | 67(5.82%) | 32(2.78%) |
