# Supplementary material for: Seed size and its rate of evolution correlate with species diversification across angiosperms
Source: PLoS Biol. 2017 Jul 19;15(7):e2002792. doi: 10.1371/journal.pbio.2002792 (PMC5536390; doi:10.1371/journal.pbio.2002792)
Supplement: S2 Table — Trait values were obtained from a 1,007 species tree where all species had data for seed size, C-value and plant height. The values are the slopes of the PGLS regressions and asterisks denote statistically significant correlations (p-value < 0.05). (DOCX) [file pbio.2002792.s020.docx]

|  | seed size | C-value | Height |
| --- | --- | --- | --- |
| seed size |  |  |  |
| C-value | 0.366* |  |  |
| Height | 0.378* | 0.074 |  |
