# Supplementary material for: Seed size and its rate of evolution correlate with species diversification across angiosperms
Source: PLoS Biol. 2017 Jul 19;15(7):e2002792. doi: 10.1371/journal.pbio.2002792 (PMC5536390; doi:10.1371/journal.pbio.2002792)
Supplement: S3 Table — Rate values were obtained from a 1,007 species tree where all species had data for seed size, C-value and plant height. The values are the slopes of the PGLS regressions and asterisks denote statistically significant correlations (p-value < 0.05). (DOCX) [file pbio.2002792.s021.docx]

|  | seed size rate | C-value rate | Height rate |
| --- | --- | --- | --- |
| seed size rate |  |  |  |
| C-value rate | 0.291* |  |  |
| Height rate | 0.535* | 0.924* |  |
