# Supplementary material for: Seed size and its rate of evolution correlate with species diversification across angiosperms
Source: PLoS Biol. 2017 Jul 19;15(7):e2002792. doi: 10.1371/journal.pbio.2002792 (PMC5536390; doi:10.1371/journal.pbio.2002792)
Supplement: S4 Table — Significant correlation are shown in bold and p-values are shown in parentheses. (DOCX) [file pbio.2002792.s022.docx]

| Macro-  evolutionary parameter | Seed size | Life Cycle |  | C-value |  | Woodiness | Height | Seed size  rate | C-value rate | Height  rate |
| --- | --- | --- | --- | --- | --- | --- | --- | --- | --- | --- |
| λ | -0.119  (0.156) | **0.202**  **(0.002)** |  | 0.183  (0.177) |  | **-0.324**  **(0.002)** | -0.157  (0.055) | **0.639**  **(<0.001)** | **0.610**  **(<0.001)** | **0.629**  **(<0.001)** |
| μ | -0.150  (0.084) | **0.198**  **(0.001)** |  | 0.215  (0.095) |  | **-0.371**  **(<0.001)** | **-0.200**  **(0.013)** | **0.544**  **(<0.001)** | **0.559**  **(<0.001)** | **0.556**  **(<0.001)** |
| r | -0.025  (0.783) | 0.105  (0.161) |  | 0.080  (0.593) |  | -0.111  (0.421) | -0.015  (0.837) | **0.587**  **(<0.001)** | **0.487**  **(0.001)** | **0.569**  **(<0.001)** |
